# Putting a Pause on Pain: Chemogenetic Silencing of NaV1.8-Positive Sensory Neurons

**DOI:** 10.1523/ENEURO.0386-23.2023

**Published:** 2023-11-03

**Authors:** Amy R. Nippert

**Affiliations:** Department of Anesthesia, Perioperative, and Pain Medicine, Stanford University, Stanford, CA 94305

**Keywords:** chemogenetics, DRG, NaV1.8, pain, sensory

The sensory system allows humans to not only explore the world but also to monitor their own bodies for injury and disease through the sensation of pain. After an injury, signals from the injured tissues travel along the nerves, entering the central nervous system through the spinal cord before going into the brain. In the acute stages, pain modulates behavior in ways that promote healing, including limiting mobility. In most cases, pain resolves as the injury heals, but, in some cases, pain persists and becomes chronic. Chronic pain is a massive issue, affecting up to 20% of Americans and leading to reduced quality of life for those affected. The factors leading to chronic pain are complex, and new approaches are needed to improve understanding of chronic pain and find effective treatments.

In the current issue of *eNeuro*, Haroun et al. (2023) use a new approach to transiently silence sensory neurons in uninjured mice as well as mouse models of acute and chronic pain using a technique known as chemogenetics. Chemogenetics is a tool used to artificially activate or inactivate cells by genetically expressing an artificial receptor. This receptor is designed to be sensitive to a specific drug. New chemogenetic options known as PSAMs (pharmacologically selective actuator modules) provide a way to directly reduce neuronal activity through modulation of ion channels (Magnus et al., 2019). This tool has distinct advantages over previous chemogenetic options, which relied on activation of indirect, complex pathways to alter neuronal signaling. In comparison, ion channels directly alter how excitable a neuron is, allowing researchers to “turn off” or “turn on” neurons with a drug. Using this tool, the authors directly and temporarily silenced sensory neurons expressing an inhibitory PSAM called PSAM^4^-GlyR with a drug called varenicline.

The authors used adeno-associated virus 9 (AAV9) to express PSAM^4^-GlyR in sensory neurons in the dorsal root ganglion (DRG), which are neurons that carry information from the periphery into the spinal cord. AAVs are viruses that can be engineered to trigger expression of a gene of interest in a particular cell type, in this case sensory neurons. This causes the neurons to express PSAM^4^-GlyR. They then administered varenicline to the mice, and looked at DRG neuronal calcium responses in vivo. They observed an 80% reduction in the number of DRG sensory neurons that had calcium increases in response to heat, indicating that the neurons were no longer activated by the heat stimulus. Next, they looked at behavioral outcomes in mice with DRG expression of PSAM^4^-GlyR. In these mice, behavioral responses to heat, cold, and mechanical stimuli were reduced only when mice had been given varenicline. There was also a reduction in hypersensitivity in models of inflammatory pain and chemotherapy induced pain.

These results are supported by a recent preprint by Perez-Sanchez et al., which showed a reduction in sensory responses with PSAM^4^-GlyR expression in DRG sensory neurons as well as reductions in pain hypersensitivity (Perez-Sanchez et al., 2023). While the reductions in pain hypersensitivity are advantageous, the authors also found reductions in responses to innocuous touch indicating a reduction in normal sensation. This loss of normal sensation, or hyposensitivity, can lead to problems such as unintentional injury.

This paper also addresses the translational problem of hyposensitivity by selectively expressing PSAM^4^-GlyR in a subtype of sensory neurons known to carry information about noxious stimuli. Different subtypes of sensory neurons are important for conveying different types of information, including information about noxious stimuli. One subtype of sensory neuron specialized for conveying painful stimulus responses is characterized by expression of the sodium channel, NaV1.8. Previous studies have deleted or permanently silenced NaV1.8 peripheral neurons and found significant reductions in pain-like behaviors. However, while these studies provided important mechanistic information, these techniques have limitations that may limit their clinical translatability. In this study, a transgenic approach was used to selectively express PSAM^4^-GlyR in NaV1.8-expressing sensory neurons to more specifically block pain information processing while leaving innocuous sensation intact. This approach also allowed temporal control over neuronal excitability, where neuronal activity was reduced only when given varenicline.

Unlike with the AAV, when mice with PSAM^4^-GlyR in NaV1.8 neurons were given varenicline they did not show reduced responses to innocuous touch, but still showed a reduction in hypersensitivity in acute and chronic pain models. These findings emphasize the need for additional research to understand the timing of NaV1.8 contributions to chronic pain as their impact may vary across pain conditions and between acute and chronic pain. Additionally, these experiments focus on immediate effects of neuronal silencing, but important questions remain about the long-term effects of repeated silencing of neurons.

In addition to their mechanistic studies, the authors also considered translational potential from their findings through their choice of the chemogenetic target PSAM^4^-GlyR and delivery through AAV9. AAVs have already been validated as a strategy for gene editing in clinical trials, providing a potential route for these findings to be used clinically. However, AAVs do have limitations, inducing unpredictable transfection rates and potential immunogenic effects. The use of PSAM^4^-GlyR also provides a clinical viable path forward, as PSAMs were designed to have high translational potential. The ligand, varenicline, is clinically approved, has excellent specificity, and the dose needed to activate PSAM^4^-GlyR is lower than the clinical dose used for smoking cessation.

Taken together, the inherent translational value and detailed mechanistic investigation from Haroun et al. (2023) provides an important foundation for future basic and clinical studies into the use of PSAMs for treating chronic pain as well as the contribution of NaV1.8 neurons to sensation of pain and innocuous touch.

## Synthesis

Reviewing Editor: Christophe Bernard, INSERM & Institut de Neurosciences des Systèmes
